# Supplementary material for: Environment DNA Reveals Fish Diversity in a Canyon River within the Upper Pearl River Drainage
Source: Animals (Basel). 2024 Aug 22;14(16):2433. doi: 10.3390/ani14162433 (PMC11350740; doi:10.3390/ani14162433)
Supplement: Supplementary file 1 [file animals-14-02433-s001.zip › Table S1.pdf]

Table S1 Genbank accession number, location information, and habitat type of the sampling sites

Sampling site	Accession number	Affiliation	Longitude(E)	Latitude(N)	Elevation(m)	Position	Habitat type
ZCH	SRR29128220	Panzhou county, Guizhou province	104.8607752	25.556465	1354.0	The upstream of dam	Eotic water
WGC	SRR29128223	Puan county, Guizhou province	104.9076871	25.546283	1275.9	The upstream of dam	Lentic water
LXK	SRR29128217	Panzhou county, Guizhou province	104.8865566	25.476417	1201.2	The upstream of dam	Lentic water
BX	SRR29128225	Panzhou county, Guizhou province	104.8828015	25.471763	1186.6	The downstream of dam	Eotic water
XTK	SRR29128221	Panzhou county, Guizhou province	104.8471738	25.431831	1163.5	The upstream of dam	Lentic water
XTX	SRR29128222	Panzhou county, Guizhou province	104.8522378	25.423945	1126.4	The downstream of dam	Eotic water
CLHD	SRR29128224	Xingyi county, Guizhou province	104.8887265	25.342771	1082.5	Lotic river without dam	Lotic water
LYZ	SRR29128216	Xingyi county, Guizhou province	104.9045946	25.313854	1035.2	The upstream of dam	Slack water
NXH	SRR29128211	Xingyi county, Guizhou province	104.8700315	25.241190	1020.9	The upstream of dam	Lentic water
MLH	SRR29128213	Xingyi county, Guizhou province	104.8511059	25.222511	1023.6	The upstream of dam	Lentic water
MLB	SRR29128214	Xingyi county, Guizhou province	104.8985086	25.202124	958.5	The downstream of dam	Eotic water
MJH	SRR29128215	Xingyi county, Guizhou province	104.8971916	25.190437	986.2	Lotic river without dam	Lotic water
MLZ	SRR29128212	Xingyi county, Guizhou province	104.9117968	25.192701	957.9	The downstream of dam	Eotic water
DAG	SRR29128218	Xingyi county, Guizhou province	104.9540050	25.140838	910.5	Lotic river without dam	Lotic water
ZJD	SRR29128219	Xingyi county, Guizhou province	104.9909766	25.049122	883.2	Lotic river without dam	Lotic water
