# Supplementary figures and images for: Environment DNA Reveals Fish Diversity in a Canyon River within the Upper Pearl River Drainage
Source: Animals (Basel). 2024 Aug 22;14(16):2433. doi: 10.3390/ani14162433 (PMC11350740; doi:10.3390/ani14162433)

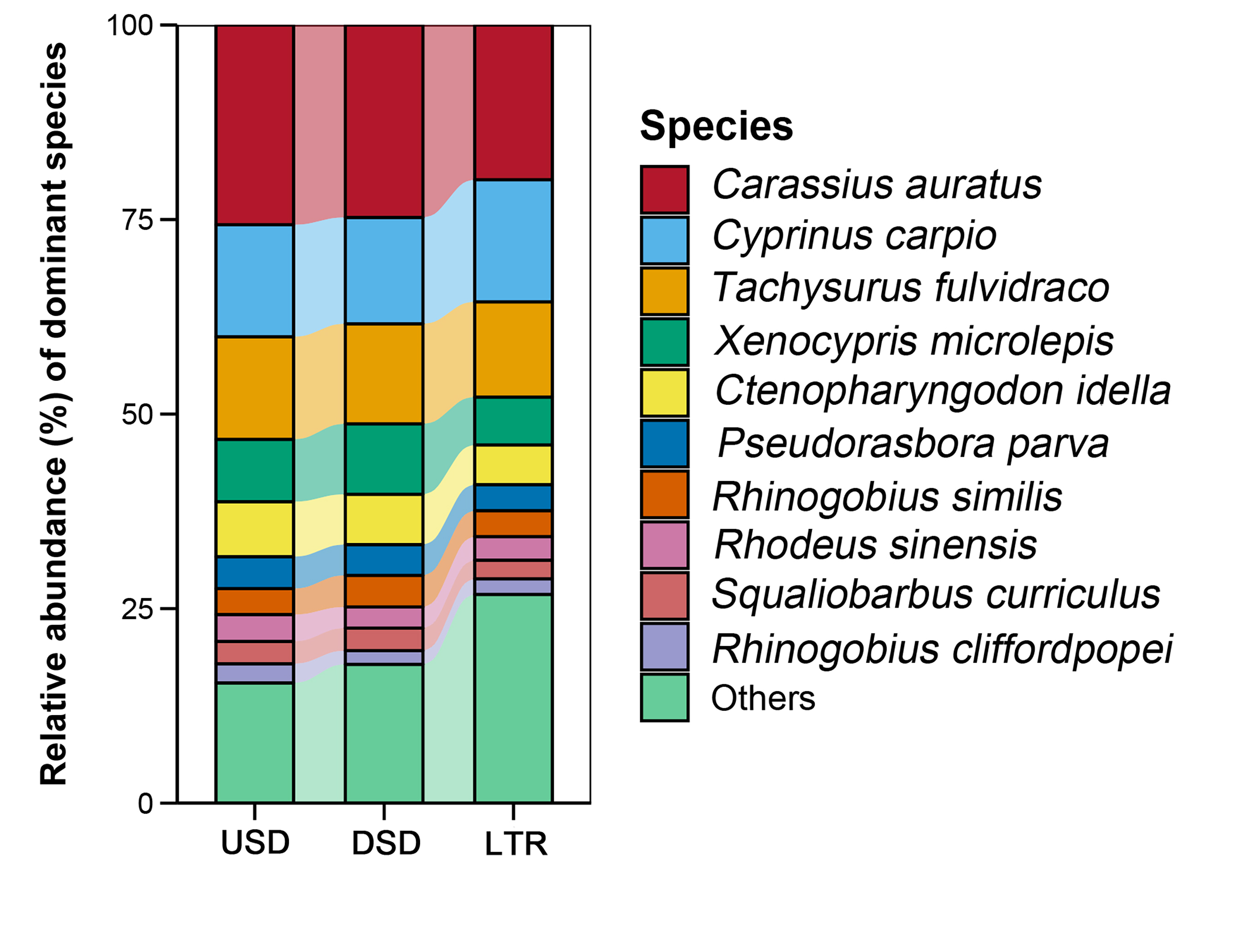

Supplement: Supplementary file 1 [file animals-14-02433-s001.zip › Figure S1.jpg]

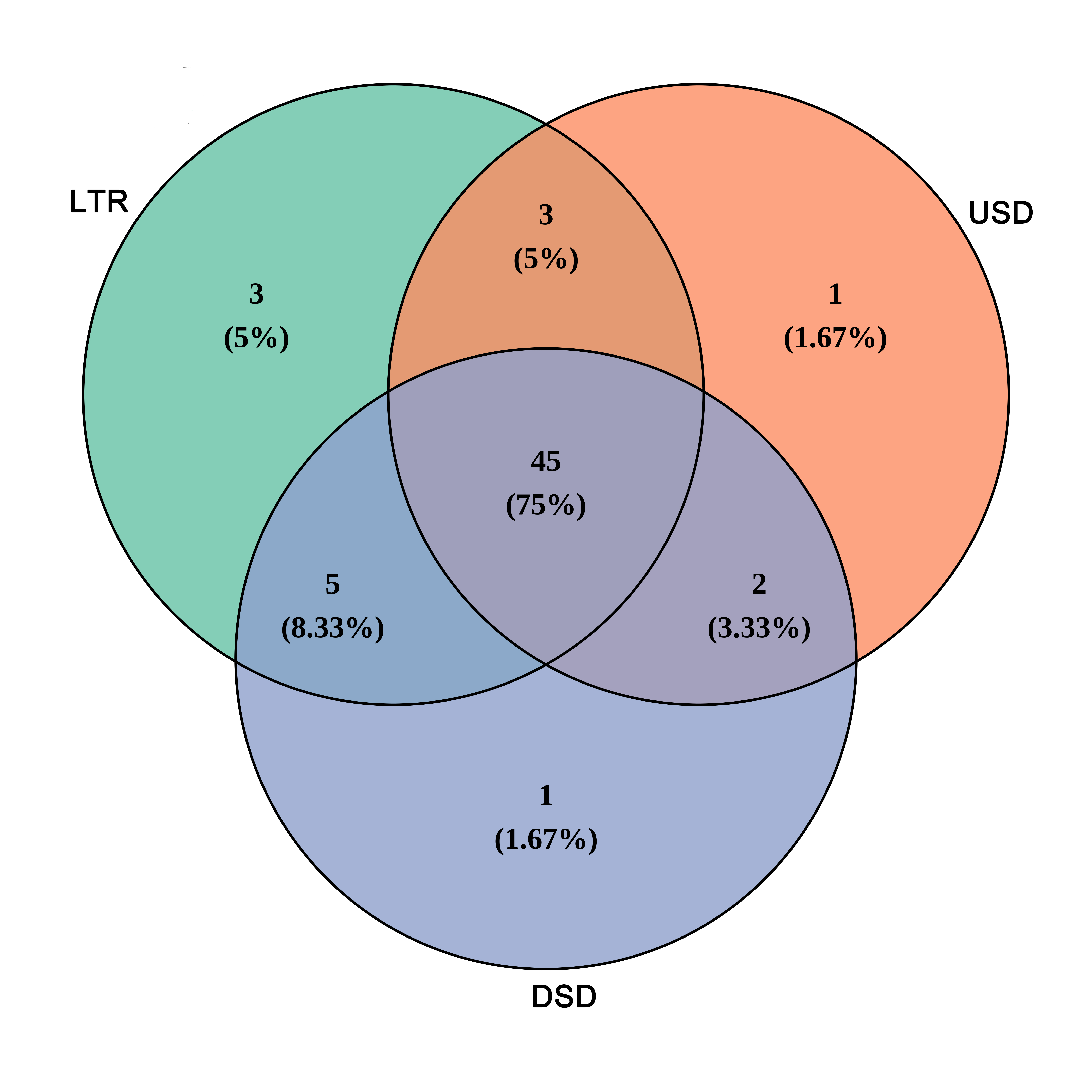

Supplement: Supplementary file 1 [file animals-14-02433-s001.zip › Figure S2.jpg]
